# Antibiotic-Loaded Coatings to Reduce Fracture-Related Infections: Retrospective Case Series of Patients with Increased Infectious Risk

**DOI:** 10.3390/antibiotics12020287

**Published:** 2023-02-01

**Authors:** Daniele De Meo, Gianluca Cera, Roberta Pica, Fabiano Perfetti, Paolo Martini, Beatrice Perciballi, Giancarlo Ceccarelli, Pietro Persiani, Ciro Villani

**Affiliations:** 1 Department of Orthopaedic and Traumatology, Policlinico Umberto I Hospital—Sapienza, University of Rome, Piazzale A. Moro, 3, 00185 Rome, Italy; 2 M.I.T.O. (Infections in Traumatology and Orthopedics Surgery) Study Group, Policlinico Umberto I Hospital, Viale del Policlinico 155, 00161 Rome, Italy; 3 Department of Public Health and Infectious Diseases—Sapienza, University of Rome, Piazzale A. Moro 5, 00185 Rome, Italy

**Keywords:** fracture, infection, osteomyelitis, coating, intramedullary nail, tibia, ORIF, hydrogel

## Abstract

Local antibiotic delivery strategies have been increasingly employed for the prevention of fracture-related infections (FRIs). The aim of this study is to evaluate the efficacy and safety of antibiotic-coated implants in the prevention of FRIs after surgical treatment in patients with increased infectious risk. A retrospective observational study has been conducted on patients with upper and lower limb fractures treated with internal fixation or prosthetic replacements, using a gentamicin coated nail (CN) and/or antibiotic-loaded hydrogel applied to the implant of choice (ALH). The study included 37 patients (20 M, 17 F), with a mean age of 63 years. The mean estimated preoperative infectious risk score was 6.4%. ALH was used in 27 cases, tibial CNs were implanted in 4 cases, and both were employed in 6 cases. The antibiotics used locally were gentamicin in 72.97% of cases (27 patients) and a combination of gentamicin + vancomycin in 27.03% of cases (10 patients). Mean follow-up was 32 months. Only one case (2.94%) showed onset of FRI at 5 months after surgery. Local antibiotic prophylaxis by coating resulted in a reduction in the incidence FRI, as compared to the estimated preoperative risk. The use of ALH allows for the choice of antibiotic; however, the application of antibiotics seems more nonuniform when applied to a nail.

## 1. Introduction

Infections are one of the major complications in orthopedics and traumatology surgery, representing an increment in hospitalization time, increased risk of reoperations or amputations, and a significant increase in spending by the healthcare system [1].

The incidence of fracture-related infections (FRIs) is highly variable, from 1.23–5% [2,3] to 33% in long-bone open fractures [4].

Implant-associated infections (FRIs and periprosthetic joint infections, PJIs) represent a therapeutic challenge for orthopedic surgeons and infectious disease specialists. Pathogens form a biofilm on the implant that creates an environment that is difficult for systemic antibiotics to penetrate; moreover, the formation of the biofilm also increases the risk of bone non-union, i.e., the body’s inability to heal a fracture and/or the loosening of the implant [5,6]. According to a recent European multicenter study in which 433 FRIs were microbiologically analyzed, the most frequently detected pathogens were Gram-positive, namely *Staphylococcus aureus* (30%), followed by other staphylococci (17%); this distribution does not vary over time [7].

Several strategies for the prevention and local reduction of infection risk have been tested in recent years. Coating systems with substances capable of preventing or reducing bacterial adhesion is a strategy that the research community has been working on for many years; however, only recently have local antibiotic prophylaxis strategies been introduced to the market [8]. The most widely used and available of these in the clinical setting include Defensive Antibacterial Coating (DAC^®^, Novagenit, Mezzolombardo, Italy) and PROtect intramedullary tibial nailing (DePuy Synthes Companies, Zuchwil, Switzerland). The former is a biocompatible gel formed by hyaluronic acid covalently bonded to polylactic acid that can be added to different types of antibiotics, limiting bacterial adhesion and releasing antibiotics in situ 72 h postoperatively [9]. The choice of the type of antibiotics is the surgeon’s responsibility, but dosage and elution properties are provided by the manufacturer. The second antibiotic prophylaxis strategy mentioned above is an intramedullary nail coated with resorbable polylactic acid (PDLLA), directly loaded at the manufacturing stage with gentamicin, capable of releasing 80% gentamicin in the first 48 h [10]. In a recent article by Baertl et al., the antibiotic susceptibility profiles of FRI pathogens was examined with respect to broadly used antibiotics and antibiotic combinations [11]. In their study, a combination of gentamicin + vancomycin was capable of covering up to 94% of pathogens, with a low resistance rate of 1.7%. The use of gentamicin in monotherapy was less successful. They concluded that approaches involving a combination of vancomycin and gentamicin, but also carbapenem, seem reasonable for use in local antibiotic therapies to treat FRIs.

Several studies in the literature demonstrate the ability of antibiotic coatings to reduce bacterial colonization and biofilm formation both in vitro [12] and in vivo [13], and to reduce the occurrence of infections when used in prophylaxis [9,14]; other studies evaluated their cost-effectiveness for the health care system [15], whether they delayed fracture healing, and their effects on prosthesis osteointegration and limb function [16].

The antibiotic-loaded hydrogel (ALH), as opposed to antibiotic-coated nails (CN), allows the surgeon’s choice of antibiotic to be added to the implant based on epidemiology or preoperative microbiological sampling, as well as the possibility of use on prostheses, but does not guarantee uniform application over the entire implant, since its application is made by the surgeon directly in the operating room and it is influenced by the shape of the implant [17].

Risk-reduction strategies and prevention play a key role in lowering FRI rates: the purpose of our study was to evaluate the prophylactic efficacy of antibiotic coatings to reduce FRI in trauma surgery. We analyzed the onset of FRIs in patients treated with one or both of the antibiotic coatings presented in the text above, their effect on fracture healing or prosthesis–bone integration, and the possible local and systemic complications related to their use, with the aim of focusing on the indications for their use in high-risk patients.

## 2. Results

### 2.1. Patients and Trauma Characteristics

The study included 37 patients, 20 males (54.05%) and 17 females (45.95%), with a mean age of 63.14 ± 24.84 years. The mean Body Mass Index (BMI) was 24.86 ± 3.91. Seven patients (18.92%) were smokers, and three patients (8.11%) were drugs addicts.

The preoperative medical history collection showed that six patients were diabetic, six had previous oncologic history, three had had an acute myocardial infarction, three had had a stroke, two had chronic kidney failure, four had chronic obstructive pulmonary disease (COPD), three had rheumatologic diseases, five had anemia of various kinds, one patient had coagulopathy, three had peripheral vasculopathy, three had dementia, and two had peptic ulcer disease. The Charlson Comorbidity Index (CCI) [18] of these patients was 3.20 ± 2.77 (Table 1).

Of the 37 patients examined, 14 (37.84%), were polytrauma patients. Thirteen patients had an open fracture defined by Gustilo—Anderson (GA) classification [19]: four GA1, five GA2, two GA3a, one GA3b, and one GA3c. The fractures we treated had articular surface involvement in 43.24% of the cases (16 out of 37 fractures). A total of 45.9% of the patients examined (17 patients) had previously existing implants in the bone segment affected by the fracture event, which were implanted at an average of 72.06 ± 81.33 months prior to fracture fixation surgery. Of these 17 patients, 64.7%, equal to 11 patients, had a hip prosthesis.

Table 2 shows the fractures we treated and the preoperative anesthesiologic risks of the patients operated upon, expressed in the American Society of Anesthesiologists (ASA) physical status classification system. It should be noted that most of the fractures treated were tibia fractures (12 cases), femur fractures (9 cases), and periprosthetic fractures (11 cases), which represent a total of 86.49% of the sample examined.

By analyzing the patients’ phenotypic characteristics, their comorbidities, the bone segments affected by the fracture events, the presence or absence of open fractures, and the patients’ anesthesiologic risks, we calculated the preoperative infectious risk of our sample according to the score of Wise et al. [20], obtaining a mean value of 5.04 ± 2.44 with a mean local surgical site infection risk of 6.4%.

### 2.2. Surgical Treatment

The average duration of the operations performed was 194.59 ± 76.21 min.

Table 3 shows the fixation devices we used to treat the fractures examined.

We used ALH alone in 27 of the 37 cases, CN alone in 4 cases, and a combination of the two in 6 cases. Antibiotics used locally were gentamicin in 72.97% of cases (27 patients) and a combination of gentamicin + vancomycin in 27.03% of cases (10 patients). Of the 37 operations, 8 were conducted by percutaneous or minimally invasive methods. In two cases it was necessary to fill the bone loss with an autologous graft, and in only one case was a synthetic graft used.

The sample’s hospitalization time averaged 20.24 ± 17.14 days. During postoperative hospitalization, 27 of the 37 patients needed at least one blood transfusion (Table 3).

### 2.3. Outcomes

Following discharge, patients underwent periodic clinical and radiographic follow-ups. The mean follow-up time of our sample was 34.41 ± 9.46 months with a minimum of 12 months. In this time frame, only one patient developed an FRI (2.70%).

The patient was a 20-year-old male, smoker and drug abuser, with a known allergy to cephalosporins, who was admitted to our emergency room following a traffic accident with a diagnosis of polytrauma, with an open femur shaft fracture (GA 3B). After placing an external fixator in damage control, he was admitted to the Intensive Care Unit and after soft tissue healing (20 days), he was treated with reamed intramedullary nailing, in which the nail was coated with ALH 5 ml supplemented with gentamicin. Five months after surgery, redness, swelling, and increased C-reactive protein developed. The CT scan showed a 3 × 6 cm bone sequestration with partial consolidation of the fracture site (two out of four cortices). The patient was therefore admitted again into the Orthopedic Department and underwent a two-staged biological chamber technique. The first stage consisted of nail removal, sequestrectomy, and debridement with positioning of antibiotic-loaded (gentamicin + vancomycin) bone cement (Vancogenx, Tecres). Intraoperative microbiology specimens (bone and periosteum, three out of five tissue biopsies), with the exception of the hardware sonication, revealed an FRI sustained by *Staphylococcus haemoliticus* that was methicillin-sensitive but gentamicin-resistant. Patient underwent targeted therapy with clindamycin 600 mg/day for 2 weeks, then another 8 weeks of outpatient oral targeted regimen. Ten weeks after surgery, the patient underwent reconstructive treatment by removing the antibiotic-loaded bone cement and replacing it with an autologous corticocancellous bone graft harvested from the iliac crest. Intraoperative samples were negative for infection. Antibiotic therapy was discontinued 2 weeks after surgery. At last follow-up, 18 months after reconstructive surgery, no clinical, radiological, or laboratory signs of infection were shown, with full functional recovery.

Of the remaining patients, only two presented postoperative complications: we found one case of delayed consolidation and one case of loosening of the hip prosthesis implant. Delayed consolidation occurred in a polytrauma patient with an open tibia fracture (GA type I) fixed with a CN, and was treated with nail dynamization. The loosening of the implant occurred after an open reduction and internal fixation of a periprosthetic femur fracture: patient underwent hip revision surgery and healed uneventfully. Intraoperative samples were retrieved during hip revision and PJI was excluded.

The remaining sample had a time to heal of 7.11 ± 2.71 months. During the outpatient visits, the patients underwent the SF-12 questionnaire for the evaluation of quality of life; this resulted in a mean MCS-12 value of 47.85 ± 11.71 and a mean PCS-12 value of 43.85 ± 10.25 (Table 4).

## 3. Discussion

This study showed that the use of antibiotic coating as prophylaxis to reduce FRI in high-infection-risk fractures has promising results. This is shown by the difference between the expected and reported infection rates in the study sample: 6.40% versus 2.70%, respectively.

There are few studies about the efficacy of antibiotic coatings in trauma surgery; all agree with the evidence here reported. In a previous review we highlighted that, while there are only two studies on this topic in the literature on trauma surgery, in the prosthetic surgery literature several relevant studies have been published [9]. In the first study about ALH, Malizos et al. [9] performed a multicenter randomized controlled prospective study, in which they compared 127 control cases to 126 cases where ALH was applied over the fixation hardware; the gel was loaded with gentamicin in 78 patients, with vancomycin 46 patients, and with vancomycin + meropenem in 2 patients. Their results showed six surgical site infections in the control group (4.7%) and no infections in the treated group. In the second study, Corona et al. treated 10 patients with post-traumatic infected bone defects of the distal femur, using a two-staged technique [21]. During the first stage, infection eradication with debridement and antibiotic-loaded bone cement was achieved; during the second stage, hydrogel loaded with vancomycin and gentamicin was used to cover the mega-prosthetic knee implant. No signs of infection were shown at last follow up. However, even if this is a study that looks at the use of antibiotic coating in a post-traumatic setting, we must highlight that is not a study that evaluates the prophylactic efficacy of ALH, but rather its use in a staged septic revision surgery to protect a mega-implant.

Open tibia fractures are known to be at high risk of infection: 1.59% in GA I, 2.99% in GA II, and 14.36% in GA III [22]. Tibia fractures were the most common injury in our population of high-risk patients (12 patients), and they were treated with a combination of the two coating systems here studied; among these patients, there were no signs of infection at last follow-up. In a previous systematic review, we highlighted relevant data about the prophylactic efficacy of CN, reporting the presence of infection in 3 cases out of 72 open tibia fractures, all of which were GA type III open fractures (7.69% of GA III open fractures) [17].

Four other studies were published after this review. Perisano et al. reported 4 surgical site infections (10.52%) and 2 (5.26%) septic non-unions among 38 open tibia fractures treated with gentamicin-coated nails [23]. Greco et al. performed an analysis of the application of CNs in 23 patients with diaphyseal open tibia fractures, compared with a control group of 23 patients [24]. They reported three superficial surgical site infections in both groups, but only one deep infection in the control group, while no bone infections in the CN group were found. Walker et al., in their report, used the ETN PROtect in 13 patients with mixed indications (open fractures, FRI, non-unions), reporting successful treatment in 11 patients (84.6%) [25]. Infectious failure occurred in the remaining patients, requiring nail removal. Patel et al. reported the largest series of 56 patients that underwent intramedullary nailing with ETN PROtect for open fractures and to treat complex revision cases [26]. They suggested the use of coated nails in high-risk patients (open fractures and those initially treated with external fixation, aseptic non-unions), reporting an incidence in those groups of 1.8%; additionally, they reported treatment failure in all of the five patients in which ETN PROtect was used to treat previously established FRI/osteomyelitis, and therefore suggested its use in those cases with caution, considering its cost.

A recent article about the cost and efficacy of coated tibia nails in GA III open fractures supported its routine use [27]. In this model, infection rates, inpatient days, theater usage, and cost have been compared between two patient cohorts of GA III open tibia fracture patients, one with and one without CN. They reported a 75% reduction in infections, 26% reduction in inpatient days, and a 10% reduction in re-operations, with a cost savings ranging from EUR 477–3263 (4–15%).

Periprosthetic fractures were also well-represented in this series (29.74%). In these cases, infections can occur more frequently; since those fractures occur near the implant, and the implant can be exposed, tested, and replaced if loosened during the fixation surgery, the risk of infection of this kind of procedure is the same as that of revision surgeries. Revision joint arthroplasty has an infection rate of 22.13% [28]. There are no specific data about the use of antibiotic coatings in periprosthetic fracture surgeries. In this series, the coating was applied to both the plates and the prosthesis, if the latter was replaced. No infection was found in any of the cases. In one case, a prosthetic loosening occurred after fixation of an apparently stable (UCS type B1) periprosthetic hip fracture, initially treated with plating and cerclage.

There are several limitations to this study. First, the population size is too small and heterogeneous with respect to the different types of fractures. Numerous other features (demographics, treatment variation, age, and BMI) may have affected the outcome as confounding variables. Even so, given the scarcity of literature on the subject, we consider it useful to present our case history. Moreover, the data presented here constitute the second-largest case report on the use of antibiotic hydrogel in traumatology, and it is the first study in which two different types of antibiotic coatings are combined. The second limitation of the study is its retrospective design and the absence of a control group of non-treated homogeneous subjects. Another limit to consider is the duration of the follow-up, even if done for a minimum of twelve months. Although the authors have considered this follow-up to be sufficiently lengthy to detect the onset of a postoperative infection, it may not be sufficiently lengthy to exclude any low-grade infections. Randomized clinical trials with lengthier follow-ups and greater sample size are therefore necessary in order to confirm the data that emerged from the present study.

## 4. Materials and Methods

### 4.1. Methods

A retrospective observational single-center study was conducted at the Orthopedic Department of Policlinico Umberto I University Hospital on patients with appendicular skeletal fractures with increased risk of infection, treated between 12/2017 and 12/2020 with osteosynthesis or prosthetic replacement, using one of the following antibiotic coatings:

- Antibiotic coating based on polylactic acid supplemented with gentamicin, affixed to the surface of the implant at the manufacturing stage. This coating appears to be commercially available only for tibial intramedullary nails (CN—PROtect Expert Tibial Nail, Synthes);
- Polylactic-acid-based rapid resorption hydrogel supplemented with antibiotic of choice, applied to the implant surface directly in the operating room. The antibiotic used in all cases was gentamicin, whether or not associated with vancomycin. Such a device can be applied to all types of implants (ALH—Defensive Antibacterial Coating, Novagenit);
- Both.

Inclusion criteria were:

- Tibia fractures treated with antibiotic-coated nail (CN);
- Fractures treated with coated implants using ALH;
- At least 12 months of clinical–radiographic follow-up;
- The presence of an increased risk of infection, defined as the presence of one or more of these factors: exposed fractures; polytrauma patients; CCI ≥ 4, in which several known infectious risk factors are assessed (advanced age, obesity, diabetes, hepatopathy, vascular disease, history of smoking, COPD).
- Exclusion criteria were:
- Patients undergoing first implant and/or revision prosthetic replacement with antibiotic cement;
- Presence of surgical site infection, superficial or deep, ongoing or previous;
- Presence of neoplastic diseases with prognosis <6 months;
- Previous diagnosis of immunodepression or immunosuppressive therapy for organ transplantation;
- Known allergy to antibiotics or coating components;
- Pregnancy or expectation of pregnancy during the study period;
- Breastfeeding.

By reviewing patients’ medical and outpatient records, several pre-, intra-, and post-operative assessment parameters were retrieved. Preoperative parameters included age, sex, Body Mass Index, comorbidities, habits, Charlson Comorbidity Index—CCI [18] (and its indicators), allergies, and ASA risk. CCI is a weighted score that takes into account several comorbidities; it predicts morality risk and the need for higher resource use. In patients with appendicular skeletal fractures, fractures were reported by anatomical district; in case of exposed fractures, exposure was classified according to Gustilo–Anderson open fracture classification, which is widely used as a tool in clinical practice to define soft tissue damage and is always reported by trauma surgeons [19]. The estimated risk of infection was calculated according to the preoperative score proposed by Wise et al. [20].

Intraoperative evaluation parameters were duration, site of surgery, type of surgery and type of implant used, type of perioperative antibiotic prophylaxis, possible use of bone grafts or substitutes, and whether the surgery was by exposure of the focus or by minimally invasive techniques. The type of coating and antibiotic used locally was also recorded for each patient.

Parameters of postoperative evaluation were length of stay, need for transfusion, incidence of postoperative infection, occurrence of other postoperative complications, and SF12 (PCS12, MCS12). Signs of implant loosening and failure of fracture consolidation were assessed by radiographs taken in at least two projections.

In the case of prosthetic replacement, the diagnostic criteria of the European Bone and Joint Infection Society [29] were used for the diagnosis of periprosthetic infections. For the diagnosis of fracture-related infections, we used the definition and criteria proposed by Matsemakers et al. [30].

### 4.2. Surgical Technique and Hydrogel Preparation

All patients performed the same pre-, intra-, and postoperative protocols regarding pain control, anesthesia, surgical wound dressing, and antibiotic prophylaxis.

Regarding the latter, all patients included in the study were given the same systemic antibiotic prophylaxis (cefazolin 2 g, 30–60 min before incision, repeated intra-operatively if surgery was >3 h in duration, followed by two postoperative doses 8 h apart), except for patients with renal failure for whom dosages were modulated according to their renal function. In contrast, patients with a cephalosporin allergy were given prophylaxis with clindamycin 600 mg; in patients with open fractures, amoxicillin + clavulanic acid 2.2 g was administered every 8 h from admission to the emergency room.

In patients with hemoglobin <8 mg/dL, blood transfusions were given, while in patients with cardiovascular disease the transfusions were given as early as Hb values <10 mg/dL.

**Figure 1:**
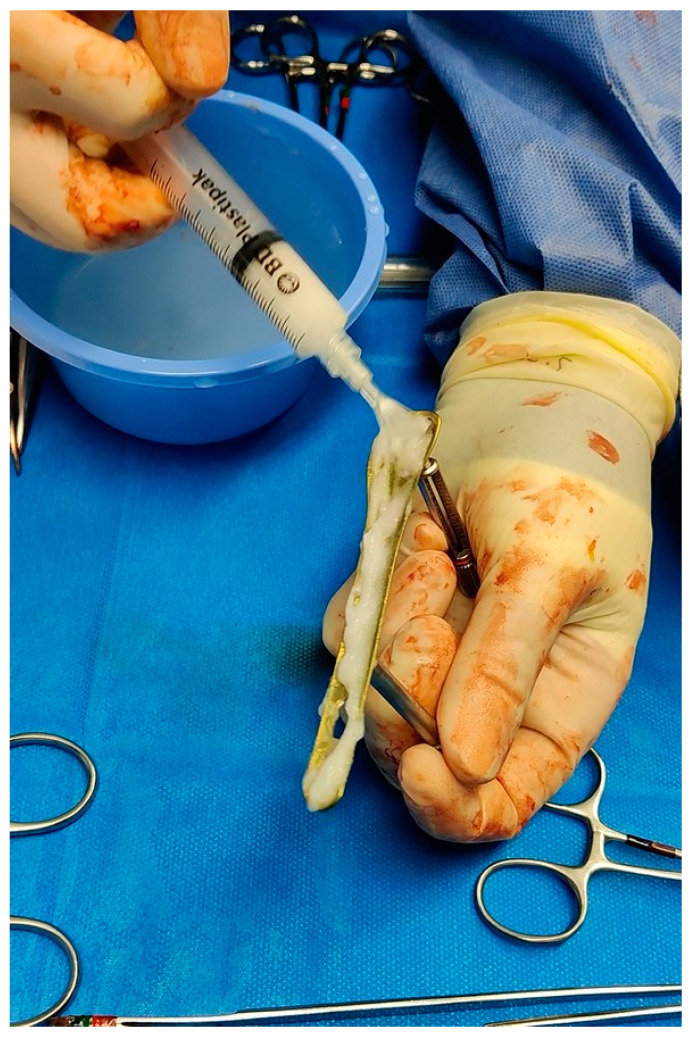
Intraoperative application of DAC gel over a distal fibula plate.

With respect to the group of patients in which the antibiotic hydrogel was used, the application of the hydrogel on the implant components took place immediately before their positioning. Gentamicin 200 mg was added to the hydrogel (5 mL) (Figure 1). In cases where the surface could not be fully coated with the content of one vial, two vials (5 mL supplemented with gentamicin 200 mg, and 5 mL supplemented with vancomycin 250 mg) were mixed together prior to use. The dosages and the elution ratio of the antibiotics into the hydrogel were those provided and tested by the manufacturer. For the patient group in which PROtect Expert Tibia Nails were used, no further preparation was needed since the coating was applied by the manufacturer during the production of the implant.

The PROtect Expert Tibial Nail (Synthes) is a nail made of an alloy of titanium, aluminum, and niobium and is coated homogeneously with an absorbable poly (d, l-lactide) matrix with gentamicin sulfate incorporated throughout, using a proprietary process [15].

In the group of patients treated with antibiotic-coated intramedullary tibia nails, the surgical technique involved the simultaneous removal of any trans-skeletal traction or external fixation previously applied as a temporary damage-control treatment. The choice of the approach (trans- or supra-patellar) was made by the surgeon on a case-by-case basis. In a subgroup of patients treated with CN, antibiotic-loaded hydrogel (5 mL with 250 mg vancomycin) was also used.

The choice of antibiotics was based upon experience with the local delivery of antibiotic polymethyl-methacrylate (bone cement), where gentamicin and vancomycin are widely applied and their efficacy is well-known [31].

Postoperative indications include early mobilization of knee and ankle, skimming load up to 3 weeks, then partial- to full-load at about 8 weeks.

### 4.3. Statistical Analysis

Continuous variables were reported using means, standard deviations, and ranges. Categorical variables were reported as an absolute value. Data were analyzed using IBM SPSS statistic 26.

## 5. Conclusions

In this retrospective study, the use of antibiotic coatings showed promising results without adverse effects. The versatility of the antibiotic-loaded hydrogel allows for a wide application in many areas but does not guarantee uniform application to the implant, whereas the antibiotic-coated nail is a preassembled system that seems to be a more practical approach when it is applicable.

## Figures and Tables

**Table 1:** Patients’ characteristics. BMI: Body Mass Index; CCI: Charlson Comorbidity Index; COPD: chronic obstructive pulmonary disease.

Patients Characteristics		
Age		63.14 ± 24.84
Gender	Male	54.05% (20)
	Female	45.95% (17)
BMI		24.86 ± 3.91
CCI		3.20 ± 2.77
Diabetes		16.21% (6)
Vascular Pathology Smoking History		8.11% (3)
		18.92% (7)
COPD		10.81% (4)
Patients’ Comorbidities (n)	0	46% (17)
	1	29% (11)
	2	11% (4)
	≥3	14% (5)

**Table 2:** Trauma characteristics. GA: Gustilo–Anderson; ASA: American Society of Anesthesiologists.

Trauma Characteristics		N (%)
Polytrauma		37.84% (14)
Gustilo–Anderson classification [19]		35.14% (13)
	GA 1	30.77% (4)
	GA 2	38.46% (5)
	GA 3a	15.38% (2)
	GA 3b	7.69% (1)
	GA 3c	7.69% (1)
ASA	1	35.14% (13)
	2	40.54% (15)
	3	21.62% (8)
	4	2.70% (1)
Intra/extra articular		
	Intraarticular	43.24% (16)
	Extraarticular	56.76% (21)
Fracture		
	Tibia	32.43% (12)
	Periprosthetic	29.74% (11)
	Femur	24.32% (9)
	Pelvis	5.41% (2)
	Humerus	2.70% (1)
	Ulna	2.70% (1)
	Radius	2.70% (1)
Pre-Op Infection Risk Score [20]		6.40%

**Table 3:** Surgical Treatment Characteristics. THA: Total Hip Artroplasty.

Surgical Treatment Characteristics		N (%)
Percutaneous/MIPO Surgery		21.62% (8)
Graft		
	No graft	91.89% (34)
	Autologous	5.41% (2)
	Synthetic	2.70% (1)
Length of surgery (minutes)		194.59 ± 76.21
Implants		
	Tibia intramedullary nail	27.03% (10)
	Femoral plate and cerclages	27.03% (10)
	THA	8.10% (3)
	THA + cerclages	5.41% (2)
	THA + plate + cerclages	5.41% (2)
	Tibia and fibula plate and screws	5.41% (2)
	Femoral intramedullary nail	5.41% (2)
	Femoral intramedullary nail + cerclages	2.70% (1)
	Femur plate and screws	2.70% (1)
	Radius plate and screws	2.70% (1)
	Pelvis plate and screws	2.70% (1)
	Shoulder endoprosthesis	2.70% (1)
	Ulna plate and screws	2.70% (1)
Coating		
	Coated nail	10.81% (4)
	Antibiotic-loaded hydrogel	72.97% (27)
	Coated ail + antibiotic-loaded hydrogel	16.22% (6)
Local antibiotic		
	Gentamicin	72.97% (27)
	Gentamicin + vancomycin	27.03% (10)
Blood transfusions (patients)		72.97% (27)
Length of stay (days)		20.24 ± 17.14

**Table 4:** Outcomes. SF-12: 12-item short-form survey; MCS-12: mental component score; PCS-12: physical component score.

Outcomes		
Infection		2.70% (1)
Other complications		
	Delay of consolidation	2.70% (1)
	Aseptic loosening	2.70% (1)
Time to heal (months)		7.11 ± 2.71
SF-12		
	MCS-12	47.85 ± 11.71
	PCS-12	43.85 ± 10.25
Follow-up (months)		34.41 ± 9.46

## Data Availability

The data presented in this study are available on request from the corresponding author.
